# Effect of ethnicity on glycaemic index: a systematic review and meta-analysis

**DOI:** 10.1038/nutd.2015.21

**Published:** 2015-07-13

**Authors:** T M S Wolever, J L Giddens, J L Sievenpiper

**Affiliations:** 1 Department of Nutritional Sciences, University of Toronto, Toronto, Ontario, Canada; 2 Keenan Research Centre of the Li Ka Shing Knowledge Institute, St Michael's Hospital, Toronto, Ontario, Canada; 3 Department of Medicine, St Michael's Hospital, Toronto, Ontario, Canada; 4 Department of Psychiatry, University of Toronto, Toronto, Ontario, Canada

## Abstract

**Objectives:**

Low glycaemic index (GI) foods are recommended to improve glycaemic control in diabetes; however, Health Canada considers that GI food labeling would be misleading and unhelpful, in part, because selected studies suggest that GI values are inaccurate due to an effect of ethnicity. Therefore, we conducted a systematic review and meta-analysis to compare the GI of foods when measured in Caucasians versus non-Caucasians.

**Methods:**

We searched MEDLINE, EMBASE and Cochrane databases for relevant articles. GI differences were aggregated using the generic inverse variance method (random effects model) and expressed as mean difference (MD) with 95% confidence intervals (95% CI). Study quality was assessed based on how well studies complied with official international GI methodology.

**Results:**

Review of 1288 trials revealed eight eligible studies, including 28 comparisons of GI among 585 non-Caucasians and 971 Caucasians. Overall, there was borderline significant evidence of higher GI in non-Caucasians than Caucasians (MD, 3.3 (95% CI, −0.1, 6.8); *P*=0.06) with significant heterogeneity (*I*^2^, 46% *P*=0.005). The GI of eight types of rice was higher in non-Caucasians than Caucasians (MD, 9.5 (95% CI, 3.7, 23.1); *P*=0.001), but there was no significant difference for the other 20 foods (MD, 1.0 (95% CI, −2.5, 4.6); *P*=0.57). MD was significantly greater in the four low-quality studies (nine comparisons) than the four high-quality studies (19 comparisons; 7.8 vs 0.7, *P*=0.047).

**Conclusions:**

With the possible exception of rice, existing evidence suggests that GI values do not differ when measured in Caucasians versus non-Caucasians. To confirm these findings high-quality studies using a wide range of foods are required.

## Introduction

The glycaemic index (GI) is conceptually defined as the incremental area under the blood glucose response curve (AUC) elicited by a portion of food containing 50 g available carbohydrate expressed as a percentage of that elicited by 50 g glucose in the same subjects. There is much evidence that GI is a relevant marker of carbohydrate quality associated with health benefits, both for people with and without diabetes.^1^ The Canadian Diabetes Association recommends the use of low-GI foods to improve glycaemic control.^2^ However, it is difficult for consumers and health professionals to obtain reliable information about the GI of specific foods because GI labeling of foods is not allowed in Canada. Health Canada recently published its opinion that including GI on food labels would be misleading and would not help consumers to make healthier food choices.^3^ A major area of concern for Health Canada is the poor accuracy and precision of the GI method. It was suggested that the GI method is inaccurate because the result obtained may depend upon the ethnicity of the subjects in whom it is measured;^3^ however, this conclusion was not based on a systematic review of the literature. Thus, our purpose was to conduct a systematic review and meta-analysis to determine whether the GI of foods differs when GI is measured in Caucasian versus non-Caucasian subjects.

## Materials and methods

The MEDLINE, EMBASE and Cochrane databases were searched from January 1981 through 16 April 2015 for relevant articles. We searched titles and keywords for (‘glycemic' OR ‘glycaemic') AND (‘index' OR ‘response') AND (‘ethnic' OR ‘African' OR ‘Asian' OR ‘Japan' OR ‘India' OR ‘Chinese'). Manual searches supplemented the electronic search strategy. No restrictions were placed on language. We included trials in which the GI values of the same foods were measured in both Caucasian and non-Caucasians in the same study. In order for a study to be included, glucose responses had to have been measured over 2 h in subjects without diabetes, incremental AUC calculated appropriately, the reference food had to be glucose or white bread, the portions of foods tested had to contain the same amount of available carbohydrate (defined as total carbohydrate minus dietary fiber, or directly measured) as the portions of the reference food, and the results for the individual foods had to be given. The only exceptions were as follows: (1) pooled results for two foods measured in Caucasians and non-Caucasians were reported in our interlaboratory study,^4^ but we report here results for each food separately; (2) one study determined the glycemic response of 50 g maltitol relative to 50 g glucose; although 50 g maltitol does not contain 50 g available carbohydrate, the study included the results that represent a glycemic response relative to that of glucose.^5^ Studies were included based on consensus of all authors. We followed PRISMA guidelines for reporting the results.^6^ No funding was received for this project and the protocol was not registered.

The mean and s.d. of the GI and number of subjects were extracted for each food separately for Caucasian and non-Caucasian subjects. For trials that reported 95% confidence intervals (CIs) rather than s.e.m. or s.d., the s.e.m. was calculated as being the CI/4. The criteria usually used to judge the quality of clinical trials, such as the Heyland Quality Score,^7^ were not considered to be relevant for judging the quality of GI studies. We judged the study quality based on whether various aspects of the International Organization for Standardization method for determining the GI of foods^8^ were reported as follows: number of subjects (1 for ⩾10 per ethnic group, 0 for <10 per ethnic group); blood sampling schedule (1 for postprandial samples at 15, 30, 45, 60, 90 and 120 min after starting to eat, 0 for any other schedule); duplicate fasting blood sample (1 for duplicate fasting blood samples or duplicate measurement of glucose in one fasting sample, 0 if no duplicate or not clearly identified); analytical coefficient of variation (CV=100 × s.d./mean) for glucose (1 for CV reported, 0 for CV not reported); repeat test of reference food (1 for ⩾2 tests of reference food per subject, 0 for single reference test or not clearly identified); reference CV being the mean of the within-individual CVs of AUC elicited by the repeated reference food tests in each subject (1 for reference CV reported, 0 for reference CV not reported); subject preparation prior to the test with respect to previous meal, fasting time, alcohol consumption, smoking and exercise (2 for >3 factors reported, 1 for 1 to 3 factors reported, 0 for no factors reported); weight of food containing 50 g available carbohydrate (1 for weight reported, 0 for weight not reported); composition of foods (fat, protein, carbohydrate and fiber) reported (1 for composition reported, 0 for composition not reported); nature of drink consumed with test meals (1 for type of drink reported, 0 for type of drink not reported). The maximum quality score was 11 points and studies with 7 or more points were considered high quality.

Data analyses were conducted using Review Manager (RevMan; (Computer program) Version 5.3. Copenhagen: The Nordic Cochrane Centre, The Cochrane Collaboration, 2014) using the inverse variance method with random effects weighting. Data were expressed as mean differences (MDs) with 95% CIs. Inter-trial heterogeneity was assessed by the Cochrane Q statistic with *α*<0.10 being considered significant, and quantified by the *I*^2^-statistic, where *I*^2^⩾50% indicates substantial heterogeneity.

## Results

### Literature search

**Figure 1:**
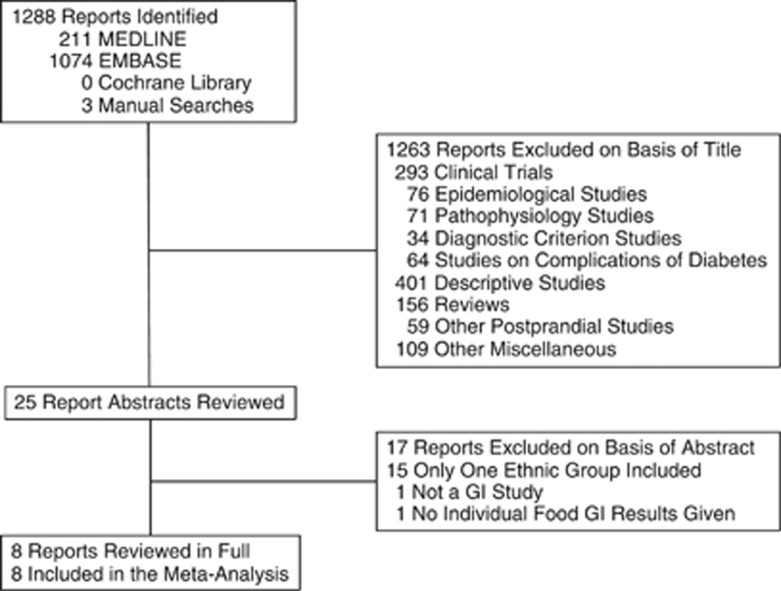
Flow of literature search.

A total of 1590 reports were identified of which 305 were removed as duplicates by the search engine, 1263 were excluded on the basis of the title and 17 were excluded after reviewing the abstract leaving 8 reports, which were reviewed in full. All eight of these reports were included in the meta-analysis (Figure 1).

### Trial characteristics

The eight studies included in the meta-analysis^5, 6, 9, 10, 11, 12, 13, 14^ reported GI values in Caucasian and non-Caucasian subjects for 28 different foods after excluding duplicates (Table 1). Seven of the eight studies included ⩾10 subjects in each ethnic group, seven used the correct blood sampling schedule, seven reported that either two fasting blood samples were obtained or the fasting sample was measured in duplicate, only two reported the CV of the glucose analytical method used, seven studies reported testing the reference food ⩾2 times in each subject but mean reference CV was reported in only three studies. All eight studies reported at least one aspect of subject preparation but only four reported ⩾3 aspects of subject preparation, four studies reported the weight of food containing 50 g available carbohydrate, one reported the amounts of fat, protein, carbohydrate and fiber contained in the portion of food fed to the subjects and five studies reported the amount and type of drink provided with the test meals. The study quality scores ranged from 5 to 10 (maximum 11), with four studies being of high quality (score ⩾7; Table 1).

### Effect of ethnicity on GI

**Figure 2:**
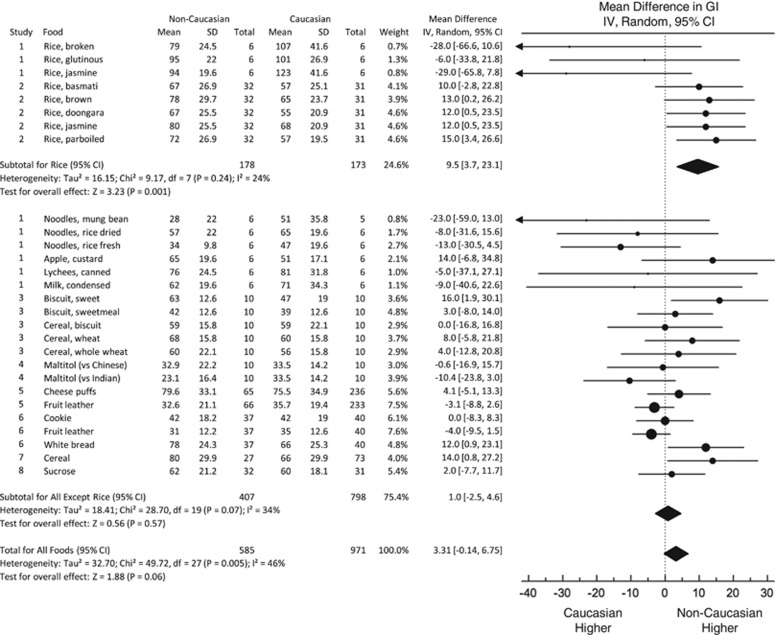
Forest plots of the effect of ethnicity on GI in participants without diabetes. Data are expressed as mean differences with 95% confidence intervals using the generic inverse variance random effects model. Pooled estimate effects are shown as diamonds. Inter-study heterogeneity was tested by Cochrane's Q statistic (*χ*^2^-test) at a significance level of <0.10 and quantified by *I*^2^, where *I*^2^⩾50% is considered to be an evidence of substantial heterogeneity. The top 8 foods are rice, the bottom 20 foods are other types of foods.

A total of 28 unique comparisons of GI were reported in eight papers among 585 non-Caucasian and 971 Caucasian subjects. Overall, the GI in non-Caucasians was a mean of 3.3 (95% CI, −0.1 to 6.8); *P*=0.06 higher than that in Caucasians; however, there was an evidence of significant heterogeneity among comparisons (*I*^2^=46% *P*=0.005; Figure 2). A *post hoc* sensitivity analysis showed that when the results of one study^10^ reporting the GI values of five types of rice were removed from the analysis, the MD in GI for the other 23 comparisons was similar in Caucasians than non-Caucasians (MD 0.8 (95% CI, −2.7 to 4.3); *P*=0.65) with evidence of moderate heterogeneity (*I*^2^=33%, *P*=0.07). If all eight comparisons of the GI of rice were analyzed separately from the other 20 comparisons, the GI of rice in non-Caucasians was significantly greater than that in Caucasians (MD 9.5 (95% CI, 3.7 to 23.1); *P*=0.001) with no evidence of significant heterogeneity (*I*^2^=24%, *P*=0.24), whereas, for other foods, the GI in non-Caucasians was similar to that in Caucasians (MD 1.0 (95% CI, −2.5 to 4.6); *P*=0.57) with evidence of moderate heterogeneity (*I*^2^=34%, *P*=0.07).

There was a significant effect of study quality on the results. In the four studies of poor quality (nine comparisons) the mean GI was significantly higher in non-Caucasians than Caucasians (MD 7.8 (95% CI, 2.4 to 13.1); *P*=0.004), whereas in the four high-quality studies (19 comparisons) the GI in non-Caucasians was not significantly different from that in Caucasians (MD 0.7 (95% CI, −3.2 to 4.7); *P*=0.76). The MD for poor-quality studies was significantly greater than that for high-quality studies (7.8 vs 0.7, *P*=0.047).

### Publication bias

**Figure 3:**
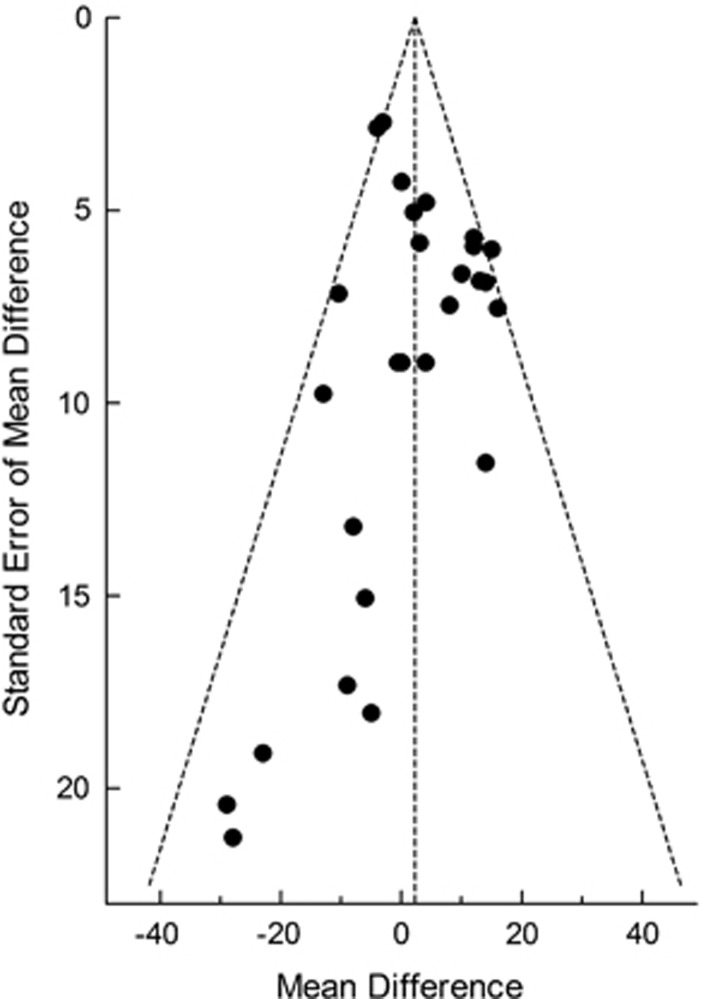
Funnel plot.

The funnel plot appeared to be asymmetrical on visual inspection (Figure 3) and this was confirmed by Egger's test (*P*<0.001) and Begg's test (*P*<0.05).

## Discussion

It has been suggested, on the basis of a non-systematic literature review, that ethnicity might affect the results of GI testing.^3^ The overall results of this systematic review comparing the GI of 28 foods in Caucasians vs non-Caucasians suggested that there was weak evidence for a small effect of ethnicity with non-Caucasians having a mean GI 3.3 higher than Caucasians (*P*=0.06). However, there was significant heterogeneity among the comparisons (*P*=0.005), which appeared to be due to one study^10^ that reported the GI values of five types of rice were greater in Chinese than European subjects. When this study was excluded from the analysis, there was no significant difference in GI between Caucasians and non-Caucasians, and there was much less evidence of heterogeneity (*P*=0.07) among the comparisons.

If the results of GI testing are affected by ethnicity, a plausible mechanism must exist to explain the effect. Here, care must be taken to distinguish between ‘glycaemic response' and ‘glycaemic index' failure to do so is common in the literature,^1, 4, 15^ but these terms describe very different things. Glycemic response, quantified by the incremental AUC, varies in different individuals based on factors such as age, sex, ethnicity, body mass index, insulin sensitivity and β-cell function. However, as GI normalizes the glycemic responses elicited by foods to the glycemic response elicited by oral glucose in the same individual, differences between subjects ought, theoretically, to be removed. Indeed, we have shown no significant heterogeneity in GI between subjects in the face of up to 10-fold differences in AUC between subjects.^16, 17^ For GI values to be affected, the factors determining the glycemic response elicited by the test food would have to differ from those for the reference food. This might occur, for example, if factors affecting the rate of digestion and/or absorption differed for the test and reference foods. A classic example of this is that the mean GI value of nine foods tested in Africans,^18^ 53.6, was similar to that for Caucasians,^19^ 54.4 (MD, −0.9 (95% CI, −5.1 to 3.4); *P*=0.70) except for milk, which was much lower in the Africans (3 vs 34) due, presumably, to their lower intestinal lactase activity. (These data were not included in the meta-analysis because the two studies were done separately and without controlling for exactly the same foods.) Another factor which could affect the absorption rate of a food, but not of oral glucose, is the degree to which a food is masticated, with more chewing leading to a smaller food particle size, a greater ratio of surface area to volume and, hence, a faster gastric emptying and/or rate of digestion/absorption.^20^ The degree of chewing of some, or all foods, varies in different people and might be culturally determined.

The paper reporting higher GI values of five varieties of rice in Chinese compared with European subjects (MD, 12.4)^10^ also reported that Chinese subjects chewed each type of rice into smaller particles than Europeans, with the difference in particle size being significant for three of the rice varieties. Although particle size data were not shown, and neither was the relationship between GI and particle size, it is tempting to speculate that the difference in GI was due to the difference in mastication. Increased chewing has been demonstrated to increase the glycemic response elicited by various foods;^21, 22^ however, the effect may be greater for some foods than others^23^ and may only occur in people with low β-cell function.^24^ In addition, the results of Kataoka *et al.*^10^ were not in accordance with those of Chan *et al.*^9^ who found that the GI of three varieties of rice tended to be higher in Caucasian than Asian subjects (MD 21). Nevertheless, with only six subjects per ethnic group, the results of Chan *et al.*^9^ carry much less weight in the meta-analysis than those of Kataoka *et al.*^10^ with 31–32 per group.

Another factor that might affect the GI value of a food in different individuals is salivary α-amylase activity (SAA). Salivary amylase is encoded for by the salivary amylase gene (*AMY1*). There is a high copy number variation of the *AMY1* gene in humans, with the number of copies of *AMY1* an individual has being directly proportional to their SAA.^25^ It has been suggested that high SAA might increase the postprandial glycemic response elicited by starch because of increased hydrolysis of starch in the mouth leading to a higher rate of starch digestion in the small intestine.^25^ If this was so, it would increase the GI value of foods determined using glucose as the reference food, because glucose needs no digestion and so its rate of absorption and glycemic response would not be affected by *AMY1* copy number variation. However, the only study to date which tested the effect of SAA on glycemic responses showed that a dextrin solution (mean degree of polymerization 40) elicited a significantly lower glucose response in subjects with high SAA than in subjects with low SAA, whereas, the glycemic response elicited by oral glucose was not different in the two subject groups.^26^ This effect was shown to be associated with a higher cephalic-phase insulin secretion in the high-SAA group after consuming dextrin.^26^ Thus, although it is possible that *AMY1* copy number variation might be associated with inter-individual variation in GI, it seems unlikely to be able to account for any difference in GI between Caucasians and non-Caucasians because *AMY1* copy number variation is highly variable within different ethnic groups; indeed it has been suggested that mean *AMY1* copy number is driven more by diet than ethnicity, being higher in populations that evolved while consuming high-starch diets.^27^ The only study included in our meta-analysis which measured SSA was the study by Kataoka *et al.*^10^ who found no significant difference in activity between Chinese and Caucasian subjects despite there being a significantly higher GI in the Chinese than the Caucasians.

We found study quality to have a significant confounding effect on the results with there being a significant effect of ethnicity on GI only in poor quality studies. However, as our assessment of study quality was not based on tools usually used to judge the quality of clinical trials, but on a novel tool developed for this meta-analysis that has not been previously validated, our results related to study quality need to be interpreted with caution. The aim of a clinical trial is to compare the effect of different treatments on an outcome or outcomes; by contrast, the aim of GI testing is to measure a food property as accurately and precisely as possible. As GI testing methodology^8^ involves cross-over studies which cannot be blinded (subjects eat real foods) with specific criteria for excluding outliers, six of the nine criteria in the Heyland Quality Score^7^ to assess risk of bias do not apply to GI studies, namely: analysis type, blinding, patient selection, comparability of groups at baseline, extent of follow-up and equality of co-interventions. Furthermore, factors known to create bias or increase random error in the results of GI testing are not among the criteria used to judge the quality of clinical trials, namely: the number of subjects,^28^ blood sampling schedule,^29^ the precision of the glucose analytical method,^4, 30^ the precision of the measure of fasting glucose,^4, 31^ the number of reference food tests,^4, 32^ within subject variation of AUC elicited by the reference food,^4^ subject preparation,^4, 33^ the amount of available carbohydrate fed to subjects,^28^ food composition^34^ and the nature of the drink consumed with the test meals.^4, 35^ Our GI methodological quality assessment did not include an assessment of randomization. As GI testing involves multiple treatments taken by each subject over a period of time, formal randomization of the order of treatments may not be desirable or ideal. For example, each subject in Henry *et al.*^11^ had eight treatments on separate occasions (five foods plus the reference food three times) over a minimum period of 2–3 weeks. To avoid bias due to time sensitive events, it is generally recommended that the reference food be tested at the beginning, middle and end of the series of foods; also it is recognized that randomization is not the only way to determine the order of testing.^28^ The official International Organization for Standardization method does not specify whether the order of tests should be randomized or not.^8^

The funnel plot demonstrated significant asymmetry, which is generally considered to indicate the presence of bias in a meta-analysis. It seems to us that, in this analysis, factors such as small studies and poor methodological quality are more likely sources of error than publication bias, but the latter cannot be ruled out.

Most, if not all, dietary advice regarding GI is based on recommending that people use low-GI foods (GI⩽55) more often and high-GI foods (GI⩾70) less often.^36^ There is no good evidence from this meta-analysis that ethnicity has an effect on the results of GI testing; however, if it did, this might result in the need for ethnic-specific cut-points for defining ‘low-GI' and ‘high-GI' based not only on the ethnicity of the consumer but also of the subjects in whom GI was measured. This would have a major implication for the regulation of GI claims on food packages. However, there is no evidence from these studies that ethnicity would disrupt the utility of GI for ranking the glycemic impact of foods; the correlation between the GI in non-Caucasians and the GI in Caucasians was highly significant (*r*=0.820, *n*=28, *P*<0.0001). For example, in the study reporting that the mean GI of 5 varieties of rice in Chinese subjects was 12 higher than in Europeans,^10^ the slope of the regression of Chinese GI on European GI was 1.01±0.18 and the correlation was excellent (*r*=0.954, *n*=5, *P*=0.012). Thus, for example, basmati rice had a lower GI than jasmine rice both in Europeans (57 vs 68) and Chinese (67 vs 80).

We conclude that, with the possible exception of rice, existing evidence suggests that food GI values do not differ when measured in Caucasians versus non-Caucasians. To confirm these findings high-quality studies using a wide range of foods are required.

## Figures and Tables

**Table 1:** Characteristics of studies included

*Authors*	*Ref/n*^a^ *test foods*	*Subjects*^b^			*Assessment of study quality*										
		*n (M:F)*	*Age*	*Origin*	*n⩾10 per group*	*Bl samp sched*	*Dup F blood*	*Anal CV*	*Repeat ref food*	*Ref CV*	*Subj prep*	*Food weight*	*Food comp*	*Drink*	*Sum*
Chan *et al.*^9^	G/9	12	22±4	A C,I,V	0	1	1	1	1	0	2	1	0	1	8
Kataoka *et al.*^10^	G/5	32:31	34±8	NZ C	1	1	1	0	1	0	1	0	0	0	5
Henry *et al.*^11^	G/5	21:26	34±11	U In	1	1	1	0	1	1	1	0	0	1	7
Pratt *et al.*^5^	G/2	15:15	29±10	U C,In	1	0	1	0	0	0	2	1	0	1	6
Wolever *et al.*^4^	GB/2	127:184	30±11	many^c^	1	1	1	0	1	1	2	0	0	1	8
Wolever *et al.*^12^	B/3	37:40	39±13	Ca ND	1	1	1	1	1	1	1	1	1	1	10
Venn *et al.*^13^	G/1	7:93	22±3	NZ C,In,K V,J,Cm	1	1	0	0	1	0	2	0	0	0	5
Venn *et al.*^14^	G/1^d^	32:31	34±8	NZ C	1	1	1	0	1	0	1	1	0	0	6

Abbreviations: A, Australia; Anal CV, coefficient of variation of glucose analytical method; B, white bread; Bl samp sched, blood sampling schedule; C, China; Ca, Canada; Cm, Cambodia; Dup F blood, duplicate fasting blood; Food comp, food composition; G, glucose; GB, glucose or white bread in multicentre trial; I, Indonesia; In, India; J, Japan; K, Korea; NZ, New Zealand; ND, not defined; RefCV, CV of area under blood glucose response food after repeated reference food tests; Repeat ref food, reference food tested ⩾2 times in each subject; Subj prep, subject preparation; U, UK; V, Vietnam.

a Reference food/number of test foods.

b Age as mean±s.d.; origin, Caucasian (line 1) and non-Caucasian (line 2/3).

c This multicentre study was carried out in 28 centers in 17 countries North America, the Caribbean, Africa, Europe, Asia, and the South Pacific.

d Duplicate values for five types of rice reported in Kataoka *et al.*^10^ were not included.

## Notes

TMSW has received research funding from the Canadian Institutes of Health Research, Canadian Diabetes Association, International Life Sciences Institute, Glycemic Index Laboratories, Inc., and Glycaemic Testing Inc. He is a part owner and employee of Glycemic Index Laboratories, Inc., and Glycaemic Index Testing, Inc. He has received consultant fees or honoraria from Bunge, Ltd., Dairy Farmers of Canada, McCain Foods, Temasek Polytechnic, Procter and Gamble, and the Glycemic Index Symbol program. His wife is an employee and part owner of Glycemic Index Laboratories and Glycaemic Index Testing, Inc. JLS has received research support from the Canadian Institutes of Health Research (CIHR), Calorie Control Council, The Coca-Cola Company (investigator initiated, unrestricted), Dr Pepper Snapple Group (investigator initiated, unrestricted), Pulse Canada, and The International Tree Nut Council Nutrition Research & Education Foundation. He has received travel funding, speaker fees, and/or honoraria from Abbott Laboratories, Pulse Canada, Canadian Sugar Institute, Dr Pepper Snapple Group, The Coca-Cola Company, Corn Refiners Association, and World Sugar Research Association. He has consulting arrangements with Winston & Strawn LLP and Tate and Lyle. His wife is an employee of Unilever Canada. JLG declares no conflict of interest.
